# Endoscopic, Endonasal Transsphenoidal Surgery for Tumors of the Sellar and Suprasellar Region: A Monocentric Historical Cohort Study of 369 Patients

**DOI:** 10.3389/fonc.2021.643550

**Published:** 2021-05-07

**Authors:** Laura Van Gerven, Zhen Qian, Anastasiya Starovoyt, Mark Jorissen, Jeroen Meulemans, Johannes van Loon, Steven De Vleeschouwer, Julie Lambert, Marie Bex, Vincent Vander Poorten

**Affiliations:** ^1^ Otorhinolaryngology, Head and Neck Surgery, University Hospitals Leuven, Leuven, Belgium; ^2^ Department of Neurosciences, Experimental Otorhinolaryngology, KU Leuven, Leuven, Belgium; ^3^ Department of Microbiology, Immunology and transplantation, Allergy and Clinical Immunology Research Unit, KU Leuven, Leuven, Belgium; ^4^ Department of Oncology, Section Head and Neck Oncology, KU Leuven, Leuven, Belgium; ^5^ Neurosurgery, University Hospitals Leuven, Leuven, Belgium; ^6^ Neurosciences, Research Group Experimental Neurosurgery and Neuroanatomy and Leuven Brain Institute, Leuven, Belgium; ^7^ Radiology, University Hospitals Leuven, Leuven, Belgium; ^8^ Endocrinology, University Hospitals Leuven, Leuven, Belgium

**Keywords:** endoscopic endonasal surgery (EES), transsphenoidal approaches, pituitary tumor, cerebrospinal fluid (CSF) leak, pituitary adenoma

## Abstract

**Background:**

The endoscopic endonasal transsphenoidal approach (EETA) is an established technique for the resection of a large variety of benign sellar and suprasellar lesions, mostly pituitary adenomas. It has clear advantages over the microscopic approach, like a superior close-up view of the relevant anatomy and the tumor-gland interface, an enlarged working angle, as well as an increased panoramic vision inside the surgical area. We have been performing the EETA for over a decade, and this study will focus on perioperative and postoperative outcomes and complications and their association with the learning curve.

**Material and Methods:**

All patients in our tertiary referral center (n = 369) undergoing an EETA for a lesion of the sellar and suprasellar region between January 1^st^ 2008 and December 31^st^ 2018 were included, and data were retrospectively retrieved from the electronic patient records.

**Results:**

Median follow-up after surgery was 55 months. Pituitary adenomas (n = 322) were the most frequent pathology. Headache (43.4%) and loss of vision (29.3%) were the most common presenting symptoms. Median procedure duration was significantly longer during the initial 5 years (106 *versus* 79 minutes; *p <*0.0001), but incidence of peri- and postoperative CSF leaks in the early years was not significantly higher. Knosp grade >2 was associated with perioperative CSF leak (*p* =0.002), and perioperative CSF leak was associated with postoperative CSF leak (*p <*0.001). Almost all cases of meningitis were preceded by a postoperative CSF leak. In 22.4% of patients, tumor recurrence required additional therapy. Perioperative (iatrogenic) mortality was 0.8%. The overall hospital stay decreased over time from an average of 7 to 5 days, and the case load increased yearly (*p* =0.015).

**Conclusion:**

The EETA is an excellent technique with complication rates comparable to or even lower than those in large microsurgical series in the literature. EETA has a significant learning curve affecting the procedure duration. Throughout the first 10 years following the transition from the microscopic approach to the EETA in our cohort, the caseload increased and hospital stay was reduced, while no increase in peri- and postoperative complications was observed.

## Introduction

Tumors with the highest incidence located in the sellar and suprasellar region are benign pituitary adenomas (1). They are derived from differentiated hormone-expressing cells located in the anterior part of the pituitary gland and are classified based on their size in microadenomas (<10 mm), macroadenomas (10–40 mm) or giant adenomas (>40 mm) or on their hormone-producing capacity (functional *versus* non-functional adenomas).

Functional adenomas (*e.g.* corticotropinomas, somatotropinomas, thyrotropinomas, and prolactinomas) generally arise from only one type of hormone-expressing cells and typically present as hypersecretory syndromes (*e.g.* Cushing’s disease, acromegaly, hyperthyroidism, and hyperprolactinemia). Prolactinomas only require surgery when medical treatment is insufficient or not tolerated (2). Usually, gonadotropinomas do not lead to hypersecretory syndromes and are diagnosed similarly to the non-functional adenomas (3). Non-functional adenomas can originate from any differentiated hormone-expressing cell, but are generally diagnosed when symptoms occur due to the size of the tumor (4). This so called mass-effect can lead to headache, hypopituitarism and/or visual field deficits. These visual symptoms arise through compression following increasing size of the longitudinal axis and typically cause hemi-anopsia. Rarely, palsy of the 3^rd^, 4^th^, and/or 6^th^ cranial nerves develops as a consequence of cavernous sinus invasion (5, 6). In very rare cases, pituitary apoplexy can occur which is characterized by sudden onset of severe headache and rapidly worsening visual field deficits or double vision caused by compression of nerves surrounding the gland. This is often followed by acute symptoms caused by lack of secretion of essential hormones. Additionally, incidentalomas in the pituitary region have become more frequent as the use of ever improving medical imaging techniques increased (7).

Less frequent benign pathologies in the sellar and suprasellar region are Rathke’s cleft cysts and craniopharyngiomas. The former are embryological remnants of the Rathke pouch and only require surgical removal in case of mass-effect or progressive growth (8, 9). The latter are congenital tumors of the central nervous system, believed to arise from residual ectoblastic cells of the craniopharyngeal duct. Craniopharyngiomas are most often located above the pituitary gland and can be resected through the endoscopic endonasal transsphenoidal approach (EETA), but often require additional radiotherapy for optimal treatment (10, 11).

Other lesions of the sellar and suprasellar region that may need to be approached for biopsy or resection are meningiomas, gliomas, and germ cell tumors, although the EETA for these lesions is less widely applied.

Historically, the gold standard for surgical removal or biopsy of all of the above pathologies has been the microscopic transsphenoidal approach. Since the year 2000, skull base tumors have increasingly successfully been approached in an endoscopic way, and our team has been among the pioneers (12–15). The EETA has clear advantages, like the increased panoramic vision inside the surgical area, resulting in better orientation for the surgeons and better close-up view of the tumor–gland interface and the relevant anatomical landmarks (16–20). Typically, neurosurgeons and otorhinolaryngologists collaborate in these skull base approaches, where they combine their knowledge and expertise during the “two nostrils–four hands” surgery. However, EETA has its limitations as well, and there are some major drawbacks coming from a microscopic approach, mainly the loss of three-dimensional vision and the longer learning curve when the surgeon is unfamiliar with endoscopic procedures.

In our tertiary referral center we have been performing the EETA for lesions in the sellar region since April 2008, after a long period of using the microscopic approach. In this retrospective, monocentric cohort study we describe our 10 year experience with the EETA and evaluate the perioperative and postoperative outcomes, with emphasis on extent of tumor resection, cerebrospinal fluid (CSF) leakage, cranial nerve damage, recurrence, and the effects of the learning curve.

## Patients and Methods

### Study Design and Data Collection

The study was approved by the Medical Ethical Committee of the University Hospitals Leuven (S63665).

**Figure 1:**
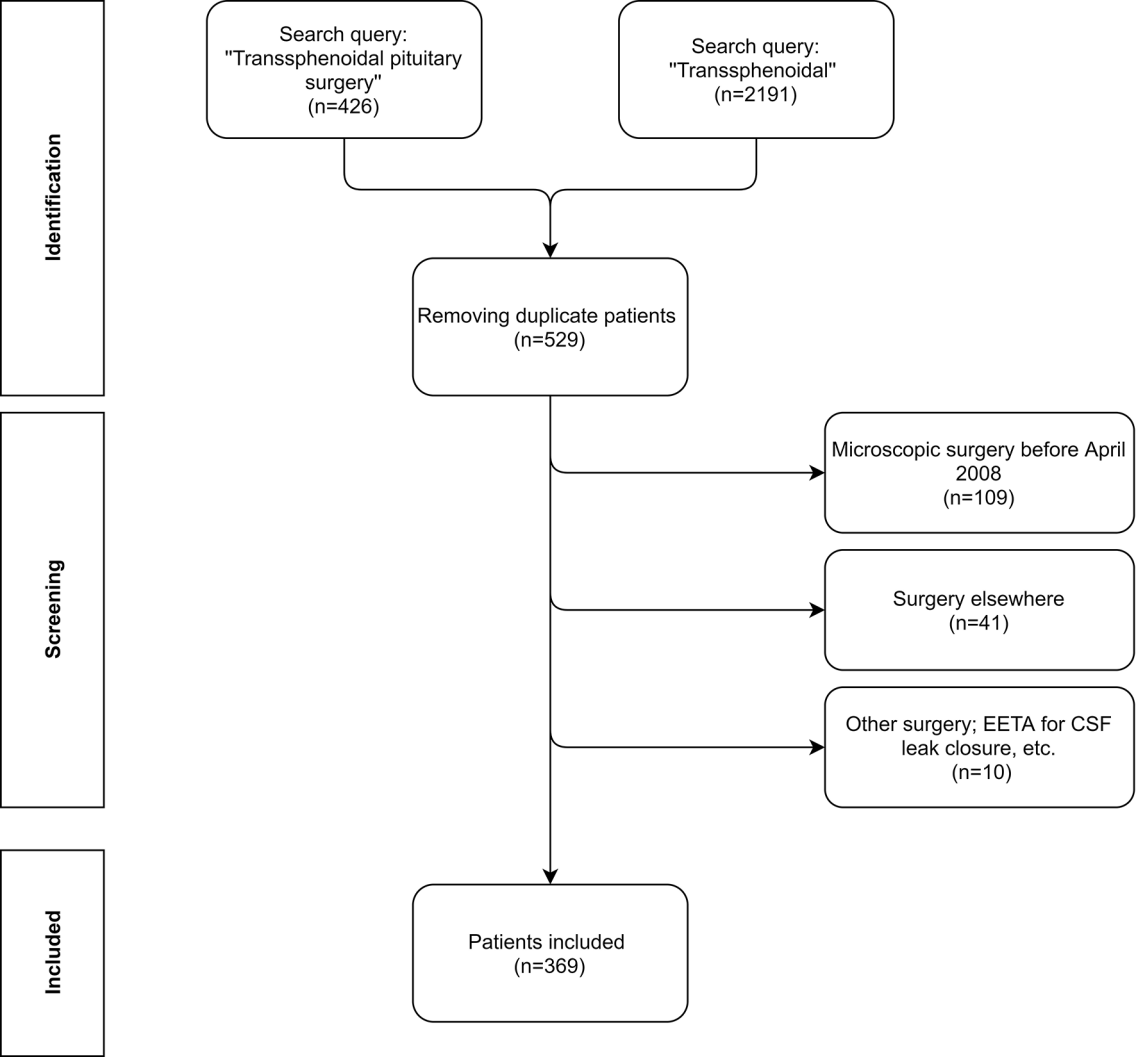
Flow diagram of the patient inclusion process. The patients were initially identified using two search queries: ‘‘Transsphenoidal pituitary surgery’’ ‘‘Transsphenoidal’’ in 2 databases; 1 was the billing file, the other the medical records. Afterwards, all of the unique patients were screened for the inclusion criteria. Note that at our hospital we do not have a separate code for CSF leak closure. Finally 369 unique patients were included in this study.

**Figure 1** depicts the flow diagram of the selection of potential patients in our electronic heath record system using two search queries between 2008 and 2018 (included). We did not include patients after 2018 to ensure a follow-up period of at least 1 year. Firstly, all patients who were billed for ‘‘Transsphenoidal pituitary surgery’’ (N = 426) were identified. Secondly all patients who had the word ‘‘Transsphenoidal’’ (N = 2191) mentioned anywhere in their electronic health record system were also identified. After removing the duplicates, 529 unique patients were found. We excluded patients that were operated *via* the microscopic approach (before April 2008), patients operated in other hospitals but in follow-up at our hospital, other types of surgery in the sellar/suprasellar region like closure of idiopathic/traumatic CSF leaks *via* a transsphenoidal approach and some other exceptions (see **Figure 1**). Subsequent removal of the patients who did not meet the inclusion criteria resulted in 369 unique patients. There was no age restriction. Patients who presented with a recurrence after surgery elsewhere or with a recurrence after previous microsurgical resection were also included.

The electronic health medical records were reviewed and analyzed for clinical, biochemical, and radiological data, procedure characteristics, perioperative complications, pathological examination of the tumor, postoperative outcomes, morbidities, and mortalities.

### Patient Work-Up and Surgical Procedure

All patients were operated under general anesthesia by a team consisting of an experienced neurosurgeon and an otorhinolaryngologist. Our standard preoperative workup included a clinical and biochemical evaluation, an MRI of the sella, and a CT scan for neuronavigation (Brainlab ^®^, Munich, Germany). If visual impairment was suspected, an ophthalmological examination was performed before surgery. All patients received perioperative antibiotic prophylaxis and a corticosteroid stress-dose. In all patients, a bilateral approach was used in three phases: the nasal, sphenoidal, and sellar phase.

After careful out-fracture of the inferior and middle turbinate with the Cottle, the natural ostium of the sphenoidal sinuses was reached *via* the paraseptal corridor (nasal phase). To enlarge the natural ostium of the sphenoidal sinuses, the inferior 3rd of the superior turbinate was removed by a monopolar cutting. The access was then enlarged by a mushroom punch and Kerrison rongeurs with caution not to damage the septal branch of the sphenopalatine artery (posterior septal artery) in patients where the use of a nasoseptal flap was anticipated. After finishing the bilateral sphenoidotomy, a posterior septectomy with resection of the rostrum and intersinus septum allowed a wide access to the face of the sella with optimal identification of the optico-carotic recess (OCR), carotic and optic protuberance on both sides, and the clival indentation (sphenoidal phase). At this point we start the two-nostril, four-handed technique to remove the sellar bone with a Kerrison punch or microdrill using a diamond burr, depending on the erosion of the bone, open the inner periosteum, and perform a meticulous endoscopy-guided resection of the tumor (sellar phase). For macro-adenoma, the inferior and lateral components of the tumor were resected before approaching the superior aspect to avoid limited vision after descent of the redundant diaphragm into the operative field. For micro-adenoma, the most challenging step was always identification of the right tumor-gland plane. In case of unclear identification, pathologic tissue which differs in color and consistency from normal pituitary tissue was removed until normal gland-tissue could be recognized.

In case of craniopharyngiomas, a resection of the solid part of the tumor and of the wall of the cystic component was attempted. In case of Rathke’s cleft cysts, a broad opening of the cyst was performed to drain the contents, and a biopsy of the wall was taken.

In the absence of perioperative complications, Spongostan^®^ (Ethicon, Edinburgh, Scotland) and Tisseel^®^(Baxter, Deerfield, Illinois, U.S.) were used to close the sellar defect. In case of a small (punctiform) intra-operative CSF-leak, a multilayer reconstruction using fascia and/or fat and a free mucosal graft in overlay were used. In case of large intra-operative CSF-leaks (macro-adenoma, malignancies), in cases in which the arachnoid was thinned out and herniated into the sella, and in case of postoperative CSF-leak, a more extensive, multilayer closure was warranted using a mucoperiosteal flap (nasoseptal/Hadad flap) in overlay instead of a free flap. Placement of lumbo-external drainage and postoperative nasal packing was not included in our standard of care but was only performed in indicated cases.

### Statistical Analysis

All statistical analysis was performed using IBM SPSS Statistics 27^®^ software or Microsoft Excel 2016. Categorical variables were expressed in frequencies and proportions. Normally distributed continuous variables were presented as means and their standard deviations, skewed continuous data as median and range. Normality was tested using Shapiro–Wilk test. Means were compared using Independent Samples T-test; medians were compared by non-parametric tests. Significance was set at *p <*0.05. One-way ANOVA was performed to investigate the association between categorical and continuous variables when appropriate, otherwise a Kruskal–Wallis test was performed. Pearson Chi-Square test was used for the association between categorical variables. Kaplan–Meier curves were calculated, and log-rank tests were subsequently performed. Recurrence-free interval was defined as time in months from date of operation until moment of either hormone suppression therapy, additional surgery, radiotherapy, or last follow-up depending on which event takes place first. Overall survival interval was defined as time in months from date of operation until date of death or last follow-up.

## Results

### Patient Characteristics

A total of 369 patients were analyzed (**Table 1**). More than half of the cohort (54.2%) was female, and the median age at surgery was 50 y (range 4–89). The median follow-up duration was 55.0 months.

**Table 1:** General patient demographics.

Patient demographics			
Number of patients		*N* = 369	%
Female		200	54.2
Median age—year		50.0	
	Range	4–89	
Median follow-up duration—months		55,0	
	Range	12–142	
Pathology			
Pituitary adenoma		322	**87.3**
	Non-hormone expressing adenoma	117	31.7
	Corticotroph adenoma	71	19.2
	Somatotroph adenoma	70	19.0
	Gonadotroph adenoma	21	5.7
	Lactotroph adenoma	14	3.8
	Thyrotroph adenoma	3	0.8
	Plurihormonal adenoma	26	7.0
Rathke cleft cyst		19	**5.1**
Craniopharyngioma		9	**2.4**
Other		19	**5.1**
Smoking		146	39.6
	Active	73	19.8
	Median pack–years	15,0	
	Range	1–200	
Comorbidities		263	71.3
	Cardiovascular	231	62.6
	Obesity	154	41.7
	Diabetes mellitus	56	15.2
	Renal	24	6.5
	Respiratory	17	4.6
	Multimorbidity	73	19.8

General demographics of the included patients.

Median pack–years are calculated for the active and non-active smokers. Cardiovascular comorbidities mainly include hypertension and hypercholesterolemia. Diabetes mellitus includes type I and type II. Renal comorbidities include chronic kidney disease and dialysis. Respiratory comorbidities include asthma, COPD, and interstitial lung diseases. Bold values are used to highlight the main groups.

Obesity was a common comorbidity (in 67%) with 154 patients classified as overweight (25 ≤ BMI < 30), 66 as class I obesity (30 ≤ BMI < 35), 23 as class II obesity (35 ≤ BMI < 40), and four as class III obesity (BMI ≥ 40). Five patients were diagnosed with multiple endocrine neoplasia syndrome.

Forty-five patients (12%) in this cohort study presented with a recurrence after previous microsurgical resection.

### Clinical and Biochemical Manifestations

Over half of the patient population presented with a non-hormonal mass effect (61.5%) (**Table 2**). Typical visual symptoms were diagnosed in 108 patients during ophthalmologic screening and included 90 patients with bitemporal hemianopsia, 15 patients with diplopia, and three with both symptoms. Eleven patients presented with pituitary apoplexy. Seventeen percent (35/201) of female patients presented with amenorrhea, and 30 patients reported sexual dysfunction. Fatigue was also a very common symptom in our cohort (151 patients).

**Table 2:** Presurgical signs and biochemical evaluation.

Presurgical signs and symptoms and biochemical evaluation				
	All		Pituitary adenoma	
	N	%	N	%
**Non-hormone mass effect**	227	61.5	185	57.3
Headache	160	43.4	127	39.3
Typical visual field defect	108	29.3	83	25.8
**Hypopituitarism**	192	52.0	172	53.4
Partial pituitary insufficiency	65	17.6	59	18.3
Panhypopituitarism	72	19.5	58	18.0
Partial pituitary insufficiency + hormone excess	53	14.4	53	16.5
Panhypopituitarism + hyperfunction	2	0.5	2	0.6
**Hormone excess**	264	71.5	238	74.0
ACTH	65	17.3	65	20.2
TSH	4	0.8	3	0.9
GH	61	16.5	61	18.9
PRL	17	4.3	17	5.3
Disconnection hyperprolactinaemia	91	24.7	66	20.4
ACTH + Stalk effect	4	1.1	4	1.2
TSH + Stalk effect	1	0.3	1	0.3
Mixed GH + TSH	1	0.3	1	0.3
GH + Stalk effect	6	1.6	6	1.9
Mixed GH + PRL	14	3.8	14	4.3

Biochemical evaluation revealed that 52% of patients had a central deficiency in at least one hormonal axis (**Table 2**). Hormone excess in this surgical series involved mostly the somatotropic axis (82 patients), followed by the corticotropic axis (69 patients).

### Tumor Characteristics and Histopathology

**Figure 2:**
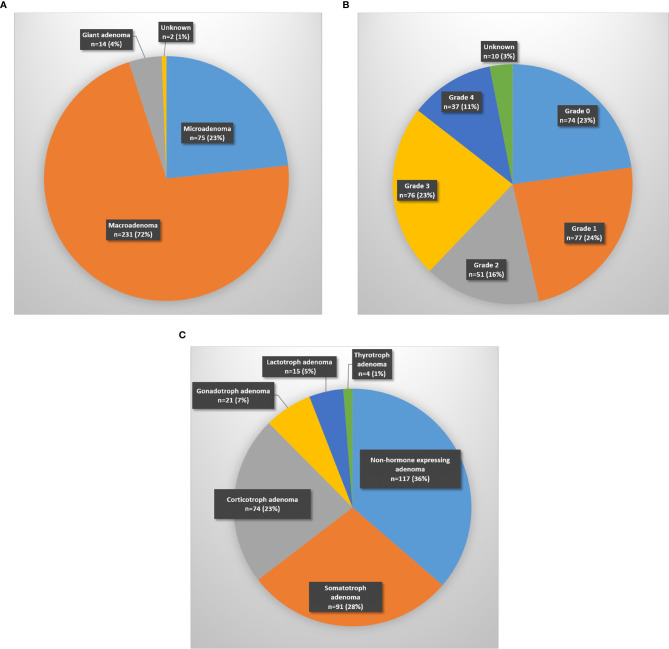
Distribution of pituitary adenomas according to **(A)** size (micro < 10 mm; macro 10–40 mm; giant > 40 mm), **(B)** Knosp classification-grade, **(C)** main hormone expression pattern.

The most frequently encountered tumors were pituitary adenomas (87.3%), followed by Rathke ‘s cleft cyts (5.1%) and craniopharyngiomas (2.4%). Most of the pituitary adenomas were macroadenomas (231/322), followed by microadenomas (75/322) and giant adenomas (14/322) (**Figure 2A**). Tumor size was not known in two. Cavernous sinus invasion by pituitary adenomas was radiologically classified using the Knosp staging system (**Figure 2B**). Non-hormone expressing adenoma was the most frequent pathological diagnosis (31.7%), followed by corticotroph adenoma (19.2%) and somatotroph adenoma (19%) (**Table 1**). The plurihormonal adenomas were further classified according to their main hormone expression pattern, and the results are visualized in **Figure 2C**. Furthermore, the EETA was used in 19 less frequent pathologies: six meningiomas, two chordomas, two oncocytomas, two plasmacytomas, two cholesterol granulomas, one germinoma, one chondrosarcoma, one neurinoma, one post-radiation sarcoma, and one leiomyosarcoma.

### Surgical Procedure and Perioperative Complications

Overall, we observed a progressive increase of EETA-procedures over the last decade (*R^2^* of 0.499; *p* =0.015) with a median yearly operated number of patients of 33 (range 15–43). Moreover, a significant reduction in operation time between the first 2 years of EETA [2008–2010: median 110.5 min, range (50; 710)] and the last three operation periods [2013–2014: median 79 min, range (40; 229); 2015–2016: median 95 min, range (20; 338); 2017–2018: median 80 min, range (20; 196)] (*p* < 0.001) could be observed (31.5; 15.5; 30.5 min respectively).

The most common perioperative complication was a CSF leak, with a significantly higher rate in the craniopharyngioma group than in the pituitary adenoma group (*p* = 0.014) (**Table 3**). There was no significant decrease in perioperative CSF leak rate over the years (*p* = 0.999). Knosp grade >2 was significantly associated with a higher perioperative CSF leak incidence (*p <*0.001) in the pituitary adenoma group.

**Table 3:** Perioperative and postoperative complications in relation to the pathology treated.

	All		Pituitary adenoma		Rathke cleft cyst		Craniopharyngioma		Other	
Median operation duration—minutes	88,0		88,5		66,0		114		92	
* Range*	20–710		25–338		40–126		59–229		20-710	
**Perioperative complications**	73	19.8%	59	18.3%	4	19%	6	66.7%	4	23.5%
Perioperative CSF-leakage	68	18.4%	55	17.1%	4	19%	5	55.6%	4	23.5%
Cavernous sinus hemorrhage	2	0.5%	2	0.6%	0	0.0%	0	0.0%	0	0.0%
Carotid artery hemorrhage	3	0.8%	2	0.6%	0	0.0%	1	11.1%	0	0.0%
**Postoperative complications**										
Postoperative CSF-leakage	27	7.3%	19	5.9%	5	23.8%	3	33.3%	0	0.0%
Diabetes insipidus										
* Transient*	42	11.4%	42	13.0%	0	0.0%	0	0.0%	0	0.0%
* Permanent*	37	10.0%	22	6.8%	9	42.9%	5	55.6%	1	5.9%
SIADH	17	4.6%	17	5.3%	0	0.0%	0	0.0%	0	0.0%
Infections	9	2.4%	6	1.9%	2	9.5%	1	11.1%	0	0.0%
Nasal obstruction	33	8.9%	26	8.1%	4	19%	0	0.0%	3	17.6
Cranial nerve damage	6	1.6%	5	1.6%	0	0.0%	0	0.0%	1	5.9%
Intracranial hemorrhage	3	0.8%	3	0.9%	0	0.0%	0	0.0%	0	0.0%
Intracranial hemorrhage and cerebral ischemia	1	0.3%	1	0.3%	0	0.0%	0	0.0%	0	0.0%
Cavernous sinus hemorrhage	1	0.3%	1	0.3%	0	0.0%	0	0.0%	0	0.0%

In two patients, profuse bleeding from the cavernous sinus impaired visualization resulting in an incomplete tumor resection.

Three patients suffered from a perioperative carotid artery hemorrhage. In only one patient, the bleeding could be controlled during the operation using Flo-Seal^®^ (Baxter, Deerfield, Illinois, U.S). The other two patients required interventional radiological therapy, which was successful in one patient.

In general, a macroscopically complete resection was achieved in 78% of the patients.

### Postoperative Complications

Median hospitalization duration was 6 days (range 1**–**62 days). Pairwise comparisons after Bonferroni correction showed that for operation period 2008–2010 (7 days, range 2**–**62), 2011–2012 (7 days, range 3**–**20) and 2013–2014 (6 days, range 2**–**59) the median hospitalization duration was significantly longer than for operation period 2017–2018 (5 days, range 1**–**17) (*p* = 0.001; *p* < 0.001; *p* = 0.004 respectively).

Transient and permanent diabetes insipidus were the most common postoperative complications (**Table 3**), followed by postoperative CSF leak and syndrome of inappropriate antidiuretic hormone secretion (SIADH).

Zooming in on postoperative CSF leak, 15 cases (4.0%) were diagnosed during the hospitalization period and 12 after hospital discharge (3.3%). Looking at factors that were potentially associated with postoperative CSF leak, surgical experience with EETA did not seem to play a role as no significant decrease was seen over the years (*p* = 0.0725). Far lateral extension (Knosp grade > 2) was also not associated with a higher incidence of postoperative CSF leak (*p* =0.875). We did see that the occurrence of perioperative CSF leak was associated with higher postoperative CSF leak incidence (*p* < 0.001). For the management of this complication, placement of a lumbo-external drainage (LED) alone was sufficient in seven patients; LED combined with surgical closure was needed in 16 patients. One patient required ventriculo-external drainage (VED) with surgical closure and another required ventriculo-peritoneal drainage (VPD) with surgical closure. One patient received only surgical closure without LED, and one patient refused a re-intervention and received only antibiotics. The average duration of temporary CSF drainage was 7 days (range 5**–**30 days). Surgical closure was always done by a multilayer reconstruction using a graft with or without a free/pedicled mucoperiosteal flap in overlay. A free muscle graft was used in 11 cases, four patients received a nasoseptal flap (overlay) combined with a muscle graft (inlay). In two patients a fat graft was used and in one patient a fascia graft.

Meningitis (eight cases) was the most frequent postoperative infection; in all but one patient this was preceded by a CSF leak in the postoperative phase. One sellar abscess developed after resection of a non-hormone expressing adenoma.

A postoperative intracranial hemorrhage was observed in four patients. In two patients symptoms of (permanent) third cranial nerve damage (diplopia and ipsilateral mydriasis) lead to the diagnosis of intracranial hemorrhage resulting in localized brain stem or cerebral ischemia. One of these patients presented with a giant adenoma with extensive cavernous sinus invasion (Knosp grade 4). The other case was a recurrence with extensive suprasellar invasion. In the other two patients the intracranial hemorrhage was found following decreased consciousness, headache, and decreased visual acuity. In total, six patients had ophthalmological confirmed cranial nerve damage after surgery. In three patients the right third cranial nerve was permanently damaged. The other three patients had transient visual problems. A last complication was severe epistaxis requiring surgical intervention in two patients. No septal perforations were observed.

### Recurrence and Overall Survival

**Figure 3:**
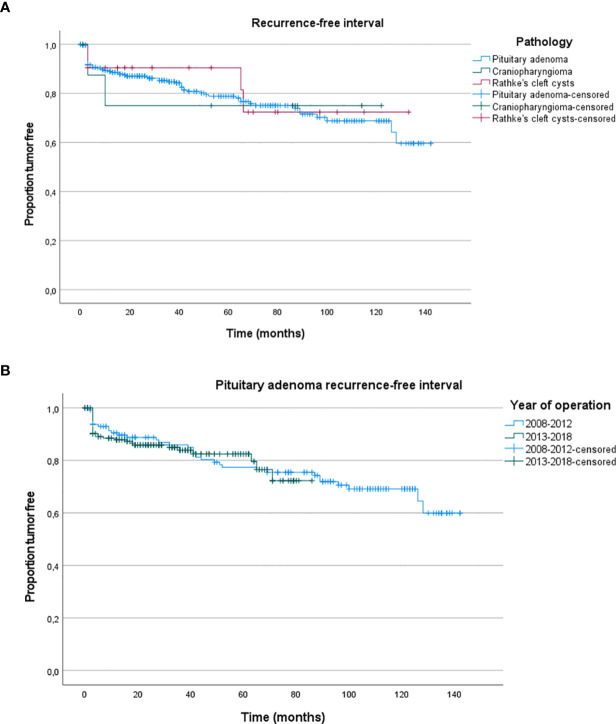
**(A)** Kaplan–Meier recurrence-free interval curves for the three most frequent histological types. **(B)** Kaplan–Meier recurrence-free interval curves for the pituitary adenomas operated during the first 5 years in comparison to the last 5 years.

At last follow-up, local control after EETA was 83.2% (**Table 4**). Focusing on the factors determining local control, previous surgical therapy and tumor size did not decrease the chance of local control (*p* =0.576, *p* = 0.462). Tumor regrowth requiring surgical reintervention was seen in 19.3% of patients with pituitary adenomas. The median time to additional surgical intervention was 15.3 months (range 1**–**96.5 months). Ultimately, 93.0% of patients had their tumor controlled either through additional surgery, radiotherapy, medication or a combination. **Table 4** displays recurrence rates and reintervention rates per disease category. In the pituitary adenoma group, most often additional therapy was needed for plurihormonal (34.6%), lactotroph (28.6%) and somatotroph (27.1%) adenomas. Corticotroph and gonadotroph adenomas had the lowest recurrence rates (16.9% and 14.3%), but with a high surgical reintervention rate (9.9% and 9.5%) as there is often no hormonal suppression therapy for these tumors. **Figure 3A** shows the Kaplan**–**Meier recurrence-free interval curves for the three most frequent types of pathology. There was no significant difference between the groups in recurrence-free interval (*p* > 0.76). **Figure 3B** shows the Kaplan**–**Meier recurrence-free interval curves for the pituitary adenomas operated during the first 5 years in comparison to the last 5 years. There was no significant difference between the first 5 years and last 5 years (*p* = 0.886).

**Table 4:** Recurrence rates and interventions in relation to the pathology treated.

Recurrence rate							
		Total		Surgical reintervention		Other interventions*	
Pituitary adenoma		62	19.3%	24	7.5%	38	11.8%
	Non-hormone expressing adenoma	15	12.8%	8	6.8%	7	6.0%
	Corticotroph adenoma	12	16.9%	7	9.9%	5	7.0%
	Somatotroph adenoma	19	27.1%	4	5.7%	15	21.4%
	Gonadotroph adenoma	3	14.3%	2	9.5%	1	4.8%
	Lactotroph adenoma	4	28.6%	1	7.1%	3	21.4%
	Thyrotroph adenoma	0	0.0%	0	0.0%	0	0.0%
	Plurihormonal adenoma	9	34.6%	2	7.7%	7	26.9%
Rathke cleft cyst		3	17.6%	3	17.6%	0	0.0%
Craniopharyngioma		2	22.2%	1	11.1%	1	11.1%
Others		16	94%	9	53%	7	41.2%

*”Other interventions” are radiotherapy and hormonal suppression therapy.

Overall, three patients died due to iatrogenic complications: one after a carotid artery hemorrhage, one due to a tonsillar herniation with a Chiari I malformation as predisposing factor, and one after an intracranial hemorrhage and unsuccessful rehabilitation.

## Discussion

In our large cohort of 369 patients that were operated by an EETA for a (para)sellar lesion between 2008 and 2018, demographics were very comparable to those of other large retrospective analyses of pituitary pathologies (1, 21). The incidence of pituitary adenomas is generally higher in females mainly due to a higher frequency and earlier signs of hyperprolactinemia in females (*e.g.* amenorrhea) (22). Not surprisingly, the majority of sellar and suprasellar lesions were pituitary adenomas (21).

Headache is a very common preoperative complaint of the patient, and our results are comparable to the literature, but unfortunately headache is also very common in the general population and therefore unspecific (23–25). Visual signs and more specifically, bitemporal hemianopsia are much more specific and also more common (28**–**100%) when there is pathology located at the (para)sellar region, according to the literature (26). Interestingly, the prevalence of visual signs in our cohort (29.3%) is located at the lower end of the reported incidence in the literature. This is unlikely to be due to smaller tumor size, as the incidence of macroadenomas in our cohort is roughly the same as in literature (27). A more plausible explanation is the fact that we only reported visual symptoms confirmed by an ophthalmologic examination.

In our experience, there was a clear learning curve reflected in duration of the surgical procedure. The EETA-procedures were initially longer, and surgical time dropped significantly going from the first two years to years 3 and 4 and then further during years 5 and 6, to then stabilize. Other authors did not observe this reduction in operative procedure duration, attributing this phenomenon to an increase in familiarity with EETA paralleling a higher acceptance of more complicated cases (28, 29). Nonetheless, we also noted a clear increase in case load over time.

Overall, the EETA is a less traumatic route to the sella as previous studies have reported (19, 30). In our cohort we did not observe any iatrogenic septal perforation, and the prevalence of epistaxis was slightly lower than reported in other series (1.25**–**11%) (31–33).

Looking at the complications, our observed rate of perioperative CSF leak (18.4%) compares favorably to what other authors reported, with incidences ranging from 15 to 25% with even reports up to 60% (29, 34–37). Younus et al. reported a decrease in perioperative CSF leak (from 60 to 33%) when the surgeon gained more experience (38). We could not observe this trend in our cohort even after including our earliest cases. This can be explained by a meticulous surgical technique ahead from the very beginning. Knosp grade is used to determine the cavernous sinus invasion in order to see preoperatively if macroscopic total resection is feasible or not (39). We found that a Knosp grade >2 is associated with higher perioperative CSF leak. A higher Knosp grade is associated with a higher invasiveness and in order to achieve a macroscopic total resection, the surgeon is required to do more extended manipulations, resulting in an increased chance of damaging the arachnoid (40, 41). Patel et al. reported that cavernous sinus invasion was not associated with perioperative CSF-leak, but did not specify their definition of cavernous sinus invasion (42).

Postoperative CSF leakage is a frequent, serious, and costly complication resulting in a higher risk of meningitis and a longer hospital stay (43, 44). The cause of this complication is either lack of recognition of a perioperative CSF leak or an incomplete closure of the leak, but small perioperative CSF leaks are not always noticeable without enhanced visualization (45).

Our rate of postoperative CSF leakage is 7.3%. Other authors report rates ranging from 1.4 to 16.9% (46, 47). Not surprisingly, there are a vast number of studies investigating how to prevent this complication (36, 48, 49). Our study shows, not surprisingly, that a perioperative CSF leak is predictive for a postoperative leak, which has been suggested in the past (50, 51). More recent literature has shown that a more intensive therapy including a perioperative lumbar drain and nasoseptal flaps in high risk patients, like those undergoing revision surgery, could be beneficial (29, 36, 37, 52, 53).

The hospitalization duration has also significantly decreased over the years. However, our hospitalization is still slightly longer (median of 5 days) than in some recently published reports, describing short-hospital-stay protocols of 3 days or less. This is mainly due to the organization of patient care in our hospital, not to a higher frequency of postoperative morbidities (54, 55).

Recurrence in pituitary adenoma occurred in around 20% of cases, which is lower than previously reported in the literature, although strongly dependent on the tumor-characteristics (24, 49, 50).

According to a recent meta-analysis, the pooled surgical remission for acromegaly is 54.8%, which is lower than the 72.9% observed in our cohort (56).

For corticotroph adenoma, Braun et al. reported that the recurrence rate ranged from 1 to 41% depending on the study, but with an average rate of 14% which is in line with the recurrence rate of our corticotroph adenoma subgroup of 16.9% (**Table 4**) (57).

Both of these types of adenomas can recur either as a macroscopically visible adenoma or as a microscopic adenoma, even undetectable on imaging, but merely based on evolution of hormonal levels. In the former case, surgery can be repeated, but in the latter, medical therapy or radiation therapy is to be considered. Lactotroph adenomas are generally not treated by surgical intervention. Only after failed medical therapy or intolerance, surgery is considered. However, surgery is often insufficient to reach complete remission. Our results in lactotroph adenomas (71.4%) are comparable to those previously reported (58–60).

## Conclusion

In this large historical cohort with long-term follow-up, EETA has proven to be a safe and efficient technique. Surgical teams that want to switch from a microscopic to an endoscopic approach should take into account the initial slightly longer operation time. However, in our series, already in the initial years, the caseload increased and hospital stay was reduced, while no increase in peri- and postoperative complications was observed. This series further adds to the body of evidence that EETA is the new gold standard for treating patients with (para) sellar lesions.

## Data Availability Statement

The data in this study are available upon reasonable request to the corresponding author. Requests to access these datasets should be directed to vincent.vanderpoorten@uzleuven.be.

## Ethics Statement

The studies involving human participants were reviewed and approved by the Medical Ethical Committee of the University Hospitals Leuven (S63665). Written informed consent from the participants’ legal guardian/next of kin was not required to participate in this study in accordance with the national legislation and the institutional requirements.

## Author Contributions

LG and ZQ: conception, data collection, drafting the article, and revising the article for important intellectual content. AS: initial data collection and revising the article for important intellectual content. MJ, JM, JLo, SV, JLa, and MB: revising the article for important intellectual content. VV: conception, data collection, drafting the article, and revising the article for important intellectual content. All authors contributed to the article and approved the submitted version.

## Conflict of Interest

The authors declare that the research was conducted in the absence of any commercial or financial relationships that could be construed as a potential conflict of interest.
